# Além da Linha de Base: Avaliação Prognóstica Dinâmica na Embolia Pulmonar Aguda

**DOI:** 10.36660/abc.20260580

**Published:** 2026-09-10

**Affiliations:** 1 Universidade Federal Fluminense Niterói RJ Brasil Universidade Federal Fluminense, Niterói, RJ – Brasil

**Keywords:** Embolia Pulmonar, Disfunção Ventricular Direita, Prognóstico

A embolia pulmonar (EP) aguda continua sendo uma das emergências cardiovasculares mais desafiadoras. A precisão diagnóstica melhorou substancialmente e as estratégias terapêuticas atuais incluem trombólise sistêmica, intervenções por cateter e equipes de resposta multidisciplinares. No entanto, a maior incerteza clínica persiste em pacientes com EP de risco intermediário a alto, nos quais a progressão natural da disfunção do ventrículo direito (VD) deve ser equilibrada com o risco de sangramento associado à terapia de reperfusão.^1^

Essa incerteza reflete uma importante limitação da avaliação de risco atual. Os modelos prognósticos contemporâneos se baseiam amplamente em variáveis medidas na admissão hospitalar.^2^ Escores clínicos, biomarcadores e exames de imagem melhoraram substancialmente a discriminação prognóstica precoce; no entanto, seu valor prognóstico deriva principalmente da avaliação inicial (PESI).^3^ A EP é inerentemente uma doença dinâmica. A adaptação do VD, a resistência vascular pulmonar, a pressão arterial sistêmica e a lesão miocárdica continuam a evoluir após o início do tratamento. Consequentemente, as informações prognósticas também devem refletir a trajetória fisiológica do paciente.

O registro RED-EP^4^ aborda precisamente essa questão clínica ainda não respondida. Em vez de introduzir novos biomarcadores, demonstra que a reavaliação de variáveis fisiológicas estabelecidas fornece informações prognósticas adicionais além da avaliação realizada na admissão. A descoberta de que a disfunção persistente do VD e a evolução temporal da pressão arterial sistólica predizem, de forma independente, desfechos adversos durante a internação sugere que as informações prognósticas continuam a se acumular durante o início da hospitalização. Consequentemente, a avaliação prognóstica deve evoluir, em vez de ser considerada completa na apresentação do paciente.

Este conceito merece atenção especial por estar firmemente fundamentado na fisiologia cardiovascular. A EP aguda impõe um aumento abrupto na pós-carga do VD por meio da obstrução vascular pulmonar e vasoconstrição secundária.^5^ A deterioração clínica depende não apenas da magnitude da obstrução embólica, mas também da capacidade do VD de se adaptar a esse desafio hemodinâmico.^6^ A disfunção persistente do VD, portanto, significa mais do que um comprometimento estrutural. Representa uma recuperação fisiológica incompleta apesar da terapia anticoagulante. Da mesma forma, a evolução da pressão arterial sistêmica pode indicar se os mecanismos compensatórios estão preservados ou progressivamente esgotados.^6^

Há mais de duas décadas, nosso grupo observou que a hipotensão sistêmica era um dos preditores mais fortes de mortalidade entre pacientes idosos hospitalizados com EP aguda.^7^ Naquela época, a hipotensão era interpretada principalmente como um marcador de instabilidade hemodinâmica. Os achados do RED-EP^4^ permitem uma interpretação mais ampla dessa observação. A hipotensão pode ser vista não apenas como uma variável prognóstica isolada, mas como a expressão clínica da falha na adaptação do VD durante o curso da doença.

Essa interpretação tem importantes implicações terapêuticas. O estudo PEITHO^8^ demonstrou que a trombólise sistêmica reduziu a descompensação hemodinâmica, mas ao custo de aumento de sangramentos graves e hemorragia intracraniana.^9,10^ Estratégias de reperfusão guiadas por cateter têm tentado melhorar esse equilíbrio, reduzindo as complicações hemorrágicas (ULTIMA;^11^ SEATTLE II;^12^ HI-PEITHO^13^). À medida que as populações envelhecem e a fragilidade se torna cada vez mais prevalente, selecionar pacientes que realmente permanecem em alto risco após o tratamento inicial torna-se ainda mais importante do que identificar o risco apenas na admissão.

A implementação prática desse conceito provavelmente dependerá de ferramentas capazes de integrar medições fisiológicas repetidas em estimativas prognósticas individualizadas. Calculadoras multimarcadoras recentes já superam os escores de risco convencionais ao incorporar informações fisiológicas longitudinais em vez de variáveis basais isoladas.^14^ O RED-EP fornece a justificativa fisiológica para essa abordagem emergente.

Portanto, o estudo RED-EP^4^ deve ser visto como algo mais do que um estudo sobre disfunção ventricular direita persistente. Ele reforça o conceito de que o prognóstico na EP aguda é um processo dinâmico. Incorporar a trajetória fisiológica do paciente na avaliação prognóstica pode refinar a predição clínica e fornecer a base fisiológica para a próxima geração de modelos prognósticos dinâmicos na EP aguda.

## References

[1] 1 Zuin M, Bikdeli B, Ballard-Hernandez J, Barco S, Battinelli EM, Giannakoulas G, et al. International Clinical Practice Guideline Recommendations for Acute Pulmonary Embolism: Harmony, Dissonance, and Silence. J Am Coll Cardiol. 2024;84(16):1561-77. doi: 10.1016/j.jacc.2024.07.044. 10.1016/j.jacc.2024.07.044 39384264

[2] 2 Creager MA, Barnes GD, Giri J, Mukherjee D, Jones WS, Burnett AE, et al. 2026 AHA/ACC/ACCP/ACEP/CHEST/SCAI/SHM/SIR/SVM/SVN Guideline for the Evaluation and Management of Acute Pulmonary Embolism in Adults: A Report of the American College of Cardiology/American Heart Association Joint Committee on Clinical Practice Guidelines. J Am Coll Cardiol. 2026;87(13):1626-710. doi: 10.1016/j.jacc.2025.11.005. 10.1016/j.jacc.2025.11.005 41712898

[3] 3 Aujesky D, Obrosky DS, Stone RA, Auble TE, Perrier A, Cornuz J, et al. Derivation and Validation of a Prognostic Model for Pulmonary Embolism. Am J Respir Crit Care Med. 2005;172(8):1041-6. doi: 10.1164/rccm.200506-862OC. 10.1164/rccm.200506-862OC PMC2718410 16020800

[4] 4 Quintana MA, Perea J, Villarreal R, Soto M, Castro M, Cortês R, et al. Disfunção Persistente do Ventrículo Direito e Evolução Hemodinâmica Predizem Deterioração Hospitalar na Embolia Pulmonar de Risco Intermediário-Alto e Alto. Arq Bras Cardiol. 2026; 123(7):e20260468. doi: 10.36660/abc.20260468 PMC13668445 42788551

[5] 5 Lyhne MD, Kline JA, Nielsen-Kudsk JE, Andersen A. Pulmonary Vasodilation in Acute Pulmonary Embolism - A Systematic Review. Pulm Circ. 2020;10(1):2045894019899775. doi: 10.1177/2045894019899775. 10.1177/2045894019899775 PMC7057411 32180938

[6] 6 Huisman MV, Barco S, Cannegieter SC, Le Gal G, Konstantinides SV, Reitsma PH, et al. Pulmonary Embolism. Nat Rev Dis Primers. 2018;4:18028. doi: 10.1038/nrdp.2018.28. 10.1038/nrdp.2018.28 29770793

[7] 7 Carvalho MRM, Oliveira GMM, Pantoja MR, Godoy PH, Luiz RR. Embolia pulmonar no paciente idoso internado. Rev SOCERJ. 2005;18(2):141-7.

[8] 8 Meyer G, Vicaut E, Danays T, Agnelli G, Becattini C, Beyer-Westendorf J, et al. Fibrinolysis for Patients with Intermediate-Risk Pulmonary Embolism. N Engl J Med. 2014;370(15):1402-11. doi: 10.1056/NEJMoa1302097. 10.1056/NEJMoa1302097 24716681

[9] 9 Goldhaber SZ. PEITHO Long-Term Outcomes Study: Data Disrupt Dogma. J Am Coll Cardiol. 2017;69(12):1545-8. doi: 10.1016/j.jacc.2017.01.027. 10.1016/j.jacc.2017.01.027 28335836

[10] 10 Barco S, Russo M, Vicaut E, Becattini C, Bertoletti L, Beyer-Westendorf J, et al. Incomplete Echocardiographic Recovery at 6 Months Predicts Long-Term Sequelae after Intermediate-Risk Pulmonary Embolism. A Post-Hoc Analysis of the Pulmonary Embolism Thrombolysis (PEITHO) Trial. Clin Res Cardiol. 2019;108(7):772-8. doi: 10.1007/s00392-018-1405-1. 10.1007/s00392-018-1405-1 PMC6584226 30564950

[11] 11 Kucher N, Boekstegers P, Müller OJ, Kupatt C, Beyer-Westendorf J, Heitzer T, et al. Randomized, Controlled Trial of Ultrasound-Assisted Catheter-Directed Thrombolysis for Acute Intermediate-Risk Pulmonary Embolism. Circulation. 2014;129(4):479-86. doi: 10.1161/CIRCULATIONAHA.113.005544. 10.1161/CIRCULATIONAHA.113.005544 24226805

[12] 12 Piazza G, Hohlfelder B, Jaff MR, Ouriel K, Engelhardt TC, Sterling KM, et al. A Prospective, Single-Arm, Multicenter Trial of Ultrasound-Facilitated, Catheter-Directed, Low-Dose Fibrinolysis for Acute Massive and Submassive Pulmonary Embolism: The SEATTLE II Study. JACC Cardiovasc Interv. 2015;8(10):1382-92. doi: 10.1016/j.jcin.2015.04.020. 10.1016/j.jcin.2015.04.020 26315743

[13] 13 Klok FA, Piazza G, Sharp ASP, Ní Ainle F, Jaff MR, Chauhan N, et al. Ultrasound-Facilitated, Catheter-Directed Thrombolysis vs Anticoagulation Alone for Acute Intermediate-High-Risk Pulmonary Embolism: Rationale and Design of the HI-PEITHO Study. Am Heart J. 2022;251:43-53. doi: 10.1016/j.ahj.2022.05.011. 10.1016/j.ahj.2022.05.011 35588898

[14] 14 Jiménez D, Dubois-Silva Á, Demelo-Rodríguez P, Ruiz-Artacho P, López-Núñez JJ, Moisés J, et al. Acute Pulmonary Embolism: A Multimarker Calculator to Predict Short-Term Outcomes. Eur Heart J. 2025;46(45):4921-9. doi: 10.1093/eurheartj/ehaf228. 10.1093/eurheartj/ehaf228 40391731

