# Supplementary material for: Além da Linha de Base: Avaliação Prognóstica Dinâmica na Embolia Pulmonar Aguda
Source: Arq Bras Cardiol. 2026 Sep 10;123(7):e2060580. [Article in Portuguese] doi: 10.36660/abc.20260580 (PMC13668450; doi:10.36660/abc.20260580)
Supplement: Translation

# Translation: Beyond Baseline: Dynamic Prognostic Assessment in Acute Pulmonary Embolism

**DOI:** 10.36660/abc.20260580i

**Affiliations:** 1 Niterói RJ Brazil Universidade Federal Fluminense, Niterói, RJ – Brazil

**Keywords:** Pulmonary Embolism, Right Ventricular Dysfunction, Prognosis

Acute pulmonary embolism (PE) remains one of the most challenging cardiovascular emergencies. Diagnostic accuracy has improved substantially, and current therapeutic strategies include systemic thrombolysis, catheter-based interventions, and multidisciplinary response teams. However, the greatest clinical uncertainty persists in patients with intermediate-to-high-risk PE, in whom the natural progression of right ventricular (RV) dysfunction must be balanced against the risk of bleeding associated with reperfusion therapy.^1^

This uncertainty reflects a major limitation of current risk assessment. Contemporary prognostic models rely largely on variables measured at hospital admission.^2^ Clinical scores, biomarkers, and imaging studies have substantially improved early prognostic discrimination; however, they derive their prognostic value primarily from the initial assessment (PESI).^3^ Acute PE is inherently a dynamic disease. RV adaptation, pulmonary vascular resistance, systemic arterial pressure, and myocardial injury continue to evolve after treatment begins. Consequently, prognostic information should also reflect the patient's physiological trajectory.

The RED-EP registry^4^ addresses precisely this unmet clinical question. Rather than introducing new biomarkers, it demonstrates that reassessment of established physiological variables provides incremental prognostic information beyond admission-based evaluation. The finding that persistent RV dysfunction and the temporal evolution of systolic blood pressure independently predict adverse in-hospital outcomes suggests that prognostic information continues to accumulate during the early course of hospitalization. Consequently, prognostic assessment should evolve rather than be considered complete at presentation.

This concept deserves particular attention because it is firmly grounded in cardiovascular physiology. Acute PE imposes an abrupt increase in RV afterload through pulmonary vascular obstruction and secondary vasoconstriction.^5^ Clinical deterioration depends not only on the magnitude of the embolic obstruction but also on the capacity of the RV to adapt to this hemodynamic challenge.^6^ Persistent RV dysfunction therefore signifies more than structural impairment. It represents incomplete physiological recovery despite anticoagulant therapy. Likewise, the evolution of systemic blood pressure may indicate whether compensatory mechanisms are preserved or progressively exhausted.^6^

More than two decades ago, our group observed that systemic hypotension was one of the strongest predictors of mortality among elderly patients hospitalized with acute PE.^7^ At that time, hypotension was interpreted primarily as a marker of hemodynamic instability. The findings of RED-EP^4^ allow a broader interpretation of this observation. Hypotension may be viewed not simply as an isolated prognostic variable, but as the clinical expression of failed RV adaptation during the course of the disease.

This interpretation has important therapeutic implications. The PEITHO trial^8^ demonstrated that systemic thrombolysis reduced hemodynamic decompensation but at the cost of increased major bleeding and intracranial hemorrhage.^9,10^ Catheter-directed reperfusion strategies have attempted to improve this balance while reducing hemorrhagic complications (ULTIMA;^11^ SEATTLE II;^12^ HI-PEITHO^13^). As populations age and frailty becomes increasingly prevalent, selecting patients who truly remain at high risk after initial treatment becomes even more important than identifying risk at admission alone.

The practical implementation of this concept will likely depend on tools capable of integrating repeated physiological measurements into individualized prognostic estimates. Recent multimarker calculators already outperform conventional risk scores by incorporating longitudinal physiological information rather than isolated baseline variables.^14^ RED-EP provides the physiological rationale for this emerging approach.

RED-EP^4^ should therefore be viewed as more than a study of persistent RV dysfunction. It reinforces the concept that prognosis in acute PE is a dynamic process. Incorporating the patient's physiological trajectory into prognostic assessment may refine clinical prediction and provide the physiological foundation for the next generation of dynamic prognostic models in acute PE.
